# Genetics of Paroxysmal Dyskinesia: Novel Variants Corroborate the Role of *KCNA1* in Paroxysmal Dyskinesia and Highlight the Diverse Phenotypic Spectrum of *KCNA1*- and *SLC2A1*-Related Disorders

**DOI:** 10.3389/fneur.2021.701351

**Published:** 2021-07-08

**Authors:** Josua Kegele, Johanna Krüger, Mahmoud Koko, Lara Lange, Ana Victoria Marco Hernandez, Francisco Martinez, Alexander Münchau, Holger Lerche, Stephan Lauxmann

**Affiliations:** ^1^Department of Neurology and Epileptology, Hertie Institute for Clinical Brain Research, University of Tübingen, Tübingen, Germany; ^2^Institute of Neurogenetics, University of Lübeck, Lübeck, Germany; ^3^Neuropediatrics Section, Hospital Universitari i Politècnic La Fe, Valencia, Spain; ^4^Genetics Unit, Hospital Universitari i Politècnic La Fe, Valencia, Spain; ^5^Institute of Systems Motor Science, University of Lübeck, Lübeck, Germany

**Keywords:** paroxysmal dyskinesia, paroxysmal kinesiogenic dyskinesia, paroxysmal exercise induced dyskinesia, *KCNA1*, *SLC2A1*

## Abstract

Paroxysmal dyskinesias (PxD) are rare movement disorders with characteristic episodes of involuntary mixed hyperkinetic movements. Scientific efforts and technical advances in molecular genetics have led to the discovery of a variety of genes associated with PxD; however, clinical and genetic information of rarely affected genes or infrequent variants is often limited. In our case series, we present two individuals with PxD including one with classical paroxysmal kinesigenic dyskinesia, who carry new likely pathogenic *de novo* variants in *KCNA1* (p.Gly396Val and p.Gly396Arg). The gene has only recently been discovered to be causative for familial paroxysmal kinesigenic dyskinesia. We also provide genetic evidence for pathogenicity of two newly identified disease-causing variants in *SLC2A1* (p.Met96Thr and p.Leu231Pro) leading to paroxysmal exercise-induced dyskinesia. Since clinical information of carriers of variants in known disease-causing genes is often scarce, we encourage to share clinical data of individuals with rare or novel (likely) pathogenic variants to improve disease understanding.

## Introduction

Paroxysmal dyskinesias (PxD) are rare movement disorders, characterized by provoked or spontaneous episodes of hyperkinetic involuntary mixed movements such as choreoathetosis, dystonia, or ballism (1, 2). There are three main PxD subtypes: paroxysmal kinesigenic dyskinesia (PKD), paroxysmal exercise-induced dyskinesia (PED), and paroxysmal non-kinesigenic dyskinesia (PNKD). Clinically, they differ from each other mainly in their duration, provocation factors, and frequency of dyskinetic attacks (Table 1) but also regarding their genetic pathology and underlying mechanism: PKD mainly results from mutations in *PRRT2* (Proline-rich transmembrane protein 2), mutations in *PNKD* (metallo-beta-lactamase domain-containing protein, formerly known as *MR-1*) may induce PNKD, and *SLC2A1* (Solute carrier family 2, member 1; known as glucose transporter 1 gene or GLUT1) is known to cause PED, among other diseases. Further genes have been linked to PxD, albeit less frequently than the already mentioned ones. Described PKD-associated genes are *SCN8A, PNKD, SLC2A1, KCNMA1, DEPDC5, KCNA1*, and *CHRNA4* (6–11). Genes associated with PNKD are *KCNMA1* (12) and *SLC2A1* (10), and the following genes have been linked to PED: *GCH1* (13, 14), *ECHS* (15, 16), and *PRRT2* (10). The aforementioned list of genes refers to isolated/predominant PxD. However, PxD can also be part of the clinical presentation in a variety of complex neurogenetic disorders (17).

**Table 1:** Phenotypic characteristics of paroxysmal dyskinesias^*^.

**Feature**	**PKD**	**PNKD**	**PED**
Trigger	(Sudden) movements	Caffeine, alcohol	Prolonged exercise, hyperventilation
Duration	Seconds to few minutes	Minutes to hours	Minutes to hours
Lateralization	Unilateral to bilateral	Unilateral to bilateral	Unilateral to bilateral
Male:female ratio	2:1	1.5:1	1:1
Age at onset	1–40 years	1–30 years	2–30 years
Frequency	Up to hundreds per day	Up to a few per day	One per day
Aura/prodromal symptoms	Sometimes	Sometimes	None
Improvement with age	Sometimes	Sometimes	Unknown
Major causative gene	*PRRT-2*	*PNKD*	*SLC2A1*
Predominant inheritance mode	Positive FH in 2/3 (3), AD/sporadic	AD (4)	AD (4)

*PKD, paroxysmal kinesigenic dyskinesia; PNKD, paroxysmal non-kinesigenic dyskinesia; PED, paroxysmal exercise-induced dyskinesia; FH, family history; AD, autosomal dominant*.

* *This table is based on Mink (5)*.

On the one hand, there is an astonishing cumulation of a few recurrent variants in PxD. For example, up to 78.5% of *PRRT-2*-positive individuals with PKD carry the frameshift mutation c.649dupC (18). On the other hand, there are reports of disease-causing variants that have occurred only in one family or only in a single individual and whose genetic evidence underpinning the pathogenicity is often limited.

In contrast, there are also secondary causes that lead to PxD such as stroke, trauma, central nervous infection, and multiple sclerosis (19), but these will not be the focus of this report.

Recently, *KCNA1* has been linked to familial PKD and PxD (7, 8, 11). In this case series, we strengthen the evidence for *KCNA1* as a causative gene in sporadic PKD and other PxD with a detailed description of the clinical phenotype of patients with two newly identified *de novo* missense mutations in *KCNA1*. We further describe a novel missense mutation in *SLC2A1* resulting in autosomal dominant PED as well as another *SLC2A1* missense mutation resulting in PED which has not been associated with this phenotype until now but has been previously published once in a different context as a *de novo* mutation in a patient with glucose transporter 1 deficiency syndrome (GLUT1-DS).

## Methods

### Study Participants

The study was performed according to local regulations and was approved by the ethics committees involved. Informed consent for publication of the clinical and genetic information was provided by all index individuals and living tested relatives. Consent for the publication of genetic information of deceased individuals was obtained from the next of kin. All study-related procedures are in accordance with the ethical standards laid down in the Declaration of Helsinki and its later amendments.

Clinical data were acquired by the investigators directly from the patient, from medical charts, or from the responsible physician. EDTA samples were taken from each available family member and DNA was extracted using standard procedures.

### Genetic Testing

We performed whole-exome sequencing (WES) in all index individuals. The exomes have been sequenced within previous research projects (individual 1: IonNeuroNet/EuroEPINOMICS, individual 3: EuroEPINOMICS) or done on clinical diagnostic basis (individuals 2 and 4). All discovered variants were validated by Sanger sequencing. The genetic variants were interpreted according to the American College of Medical Genetics and Genomics (ACMG) standards and guidelines for the interpretation of sequence variants. The fulfilled criteria are listed in brackets with the abbreviations used in the reference literature (20).

## Results

### Individual #1: *KCNA1* p.Gly396Val (c.1187G>T)

#### Clinical Description

This 49-year-old man has been followed up from 2003 to 2020. He started having attacks of PKD at the age of 10 years. The paroxysmal events were usually provoked by sudden movements, for example, at the start of a 100-m run, or when he was suddenly called while waiting in the waiting room. He reported having twisting and cramping movements of the right arm and leg which rarely spread to the left side of the body. Sometimes, the symptoms were so severe that he could no longer hold himself upright. The attacks lasted between 20 and 60 s and there were no prodromes, for example, sensory phenomena and no alteration of consciousness. He also did not notice any vertigo or the feeling of being drunk during the episodes. Other triggers such as consumption of alcohol, caffeinated drinks, or certain food or the feeling of being cold or fatigue were denied. In case of high fever, the attacks were more frequent, and on rare occasions, they could also occur at rest. The family history was negative for neurological disorders.

Carbamazepine had reduced attacks but was not tolerated because of side effects. Gabapentin was not helpful. The patient did not want to try other medications. The neurological examination revealed an asymmetrical right accentuated arm tremor but was otherwise normal (in particular no ataxia or myokymia, electromyography was not performed). EEG and MRI findings were normal. Genetic findings will be presented with the ones of individual #2.

### Individual #2: *KCNA1* p.Gly396Arg (c.1186G>C)

#### Clinical Description

This 17-year-old woman suffers from idiopathic generalized epilepsy and PxD. She had her first epileptic seizure at the age of 18 months when she suffered from atonic seizures with a sudden loss of tone for a few seconds with falling. She started to have generalized tonic–clonic seizures (GTCS) at the age of 6 years. At the same age, she had a febrile convulsive status epilepticus requiring intubation. Her first episodes with abnormal involuntary choreatiform movements started at the age of 12 years. Both legs and arms and facial muscles were affected. The paroxysmal events lasted 3 to 5 s, occurred two to three times per day, and were triggered by emotional stress, not by sudden movements or exercise.

Development was described as normal, yet when she was 6 years old, she was diagnosed with attention deficit hyperactivity disorder and with a mild difficulty in expressive language. There have been no neurologic disorders in the family.

She was first treated with valproate which was withdrawn due to lack of seizure control. Her second antiseizure drug was topiramate which was stopped due to anhidrosis. Levetiracetam led to an increase in seizure activity so the medication was exchanged with oxcarbazepine which was very effective. When she suffered from a febrile convulsive status epilepticus at the age of 6 years, valproate was added again and taken together with oxcarbazepine until the age of 15. Since paroxysmal dyskinesia did continue, she additionally received brivaracetam from the age of 14 years on. Only when lacosamide, a third anticonvulsive drug, was added 1 year later (in exchange for valproate), she became free of paroxysmal dyskinetic events. Her current treatment (last update: February 2021) is oxcarbazepine 2 × 600 mg (19.05 mg/kg body weight per day), brivaracetam 2 × 50 mg (1.59 mg/kg body weight per day), and lacosamide 2 × 150 mg (4.76 mg/kg body weight per day).

Physical examination did not reveal any signs of ataxia, impaired coordination, or tremor. Myokymia was observed clinically. Further significant findings were generalized hyperhidrosis and acne vulgaris.

A 6-h EEG recording after sleep deprivation (10–20 standard EEG recording, age: 15 years and 9 months) revealed intermittent generalized theta activity (4–6/s) and high amplitude delta activity (3–4/s) predominantly in stage I sleep as well as generalized 3/s spike-wave activity of up to 1 s duration during wakefulness without any clinical correlate. A small subcortical area of gliosis/demyelination right frontobasally was found in the MRI of the brain and interpreted as an unspecific finding.

#### Genetic Testing and Variant Interpretation (KCNA1) of Individual #1 and #2

**Figure 1:**
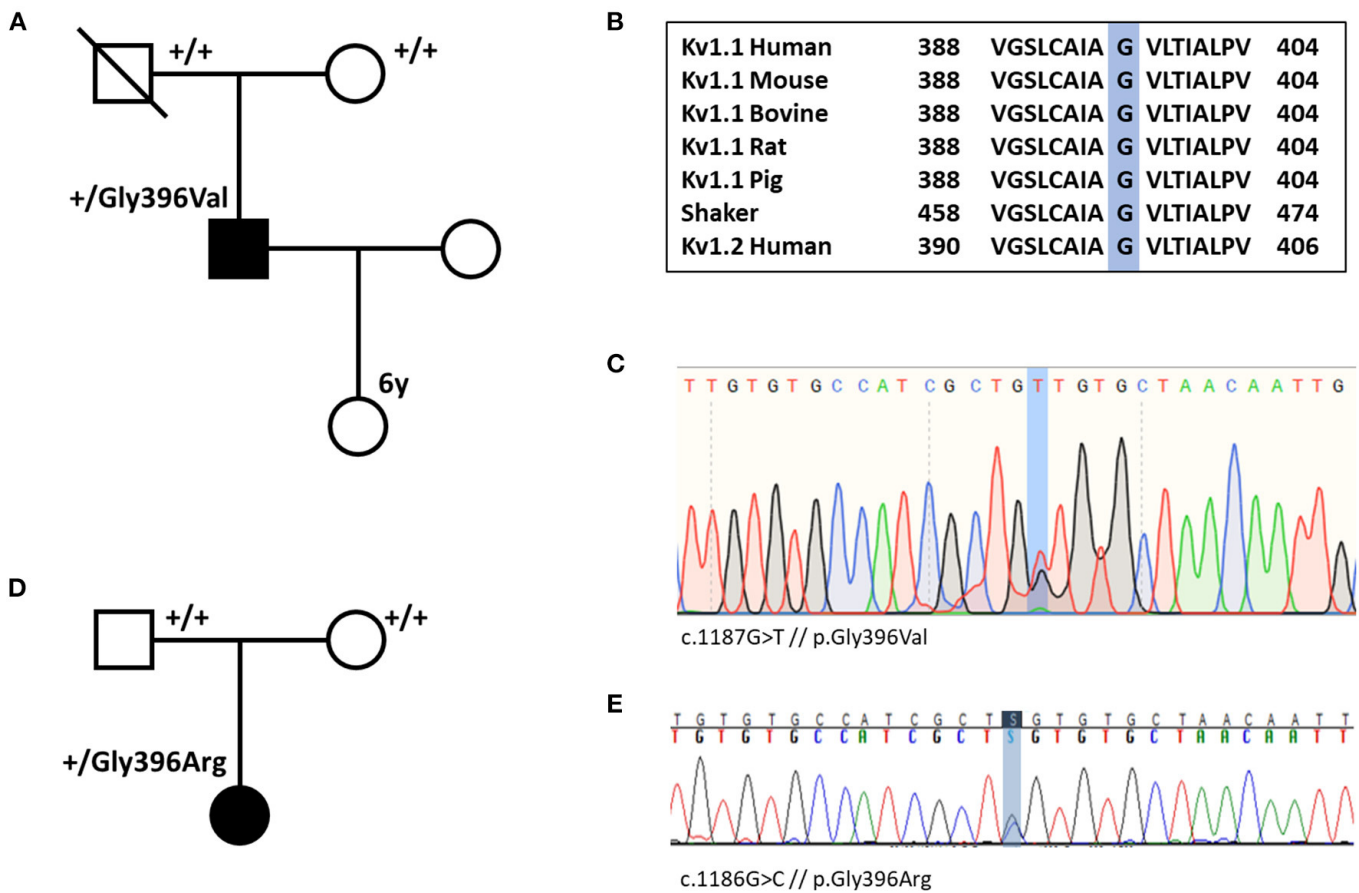
**(A)** Pedigree of individual #1 shows that the variant p.Gly396Val (p.G396V) is *de novo*. **(B)** Amino acid sequence alignment of Kv1.1 subunits across several species and human Kv1.2 subunit demonstrates conservation of the Gly396 residue. **(C)** Nucleotide sequence with heterozygote substitution of G with T (c.1187G>T) in individual #1. **(D)** Pedigree of individual #2 demonstrates *de novo* status of the variant. **(E)** Nucleotide sequence with heterozygote substitution of G with C in individual #2 (c.1186G>C).

Whole-exome sequencing for individual #1 was performed in 2012. At that time, no disease-causing variant could be identified (including analysis of *PRRT2* and *SLC2A1*). Exome data reanalysis of six exome-negative patients with paroxysmal movement disorders from our local patient database in 2020 revealed the variant p.Gly396Val in *KCNA1* (Figure 1). In the remaining five exomes (three samples of individuals with PKD and two samples of individuals with PED), we did not find any significant variants. Individual #2 received whole-exome sequencing on a diagnostic basis in 2020 where the variant p.Gly396Arg was detected.

Both variants are confirmed to be *de novo* and they were not listed in gnomAD (https://gnomad.broadinstitute.org/) (21). The amino acid change is located in the pore-forming transmembrane segment S6. Alignment of Kv1 channels across species, shaker, and Kv1.2 (Figure 1) indicates that the Gly396 residue is highly conserved among all species and tested channels. Only two familial variants in *KCNA1* causing PKD have been described so far. While p.Asn255Lys sits in the S3-helix, p.Leu319Arg is located in an intracellular linker that connects the voltage-sensing domain to the pore (between S4 and S5) (22, 23). Both variants are in a completely different region compared with our variants. According to the ACMG criteria, the variants of individual #1 and #2 are predicted to be likely pathogenic [PM1, PM2, PM6, PP2, PP3; the abbreviations refer to the ACMG reference publication (20)]. All 21 *in silico* prediction tools listed on VarSome (free version) predict a deleterious effect. Furthermore, electrophysiological studies in *Xenopus* oocytes at homolog positions in the *KCNA2* gene (Gly398Cys) and in the well-studied *shaker* channel (Gly466Ala and Gly466Trp) revealed loss-of-function defects for all three variants (24–26).

### Individual #3: *SLC2A1* p.Met96Thr (c.287T>C)

#### Clinical Description

This 26-year-old woman has suffered from PED since childhood. Symptoms emerged when she began to be able to do prolonged exercise like hiking with the family or playing table tennis. Predominant symptoms of PED were dystonia of the legs that occurred after or during prolonged exercise and up to three to four times per week. After the interruption of physical activity and intake of glucose (dextrose), she was able to move on after 10–15 min. Regular intake of carbamazepine retard 15 mg/kg per day led to a reduction of the attack frequency to three to four episodes per 6 months.

At the age of 10 years, she presented with an episode which was classified as an attack of hemiplegic migraine: in school, she suffered from a sudden headache with subsequent paresis of the left leg, followed by twitches of the left leg and stiffness of the left arm, which was rotated inwards. Consciousness and awareness have been normal for the whole episode; there was no tongue bite, no defecation, and no enuresis. The speech was blurred. Her symptoms were regressing during the transport to the hospital. EEG revealed delta slowing of the right hemisphere. The paresis faded after 1 to 2 h. A hemiplegic migraine attack had been suggested. Such an episode did not recur. Since she did not feel the episodes of PED to be disturbing, she stopped taking carbamazepine between 11 and 13 years of age.

Mild coordination impairment was observed during development and she needed logopedic support to improve her speech skills. She had a learning disability and physical examination revealed mildly reduced fine motor skills. EEG repeatedly showed increased non-lateralized theta rhythms.

**Figure 2:**
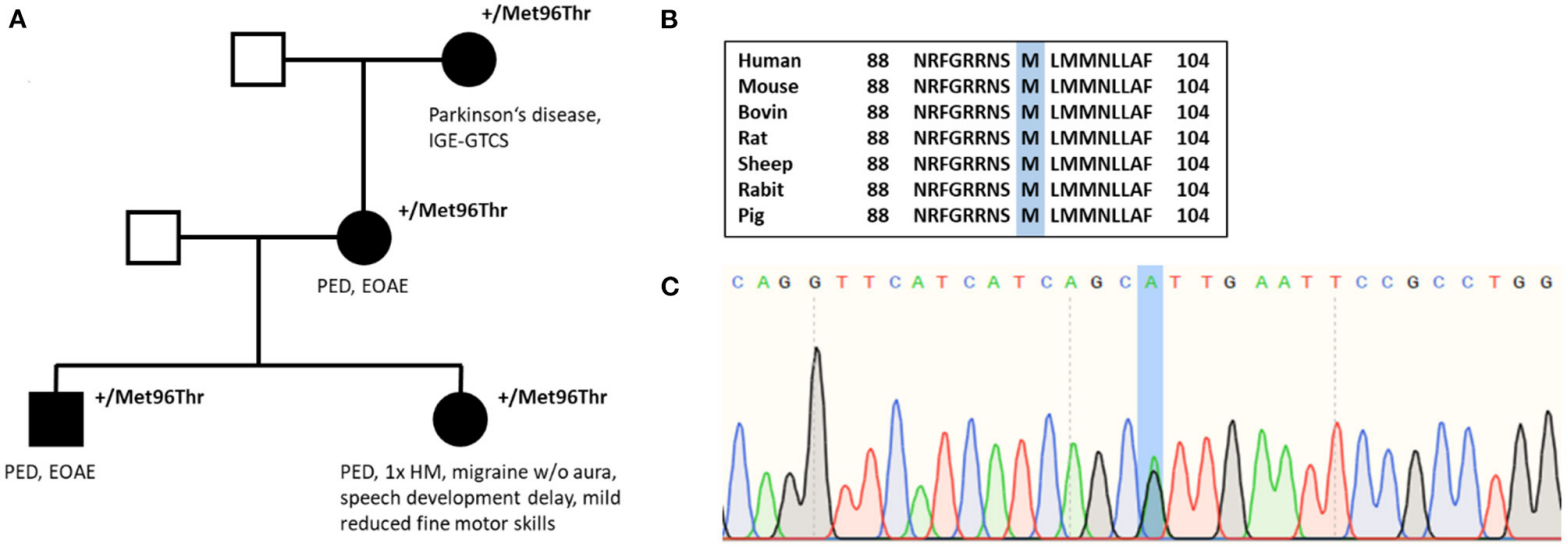
**(A)** Pedigree of individual #3 shows autosomal dominant inheritance. **(B)** Amino acid sequence alignment of *SLC2A1* across several species demonstrates conservation of the Met96 residue. **(C)** Nucleotide sequence with substitution of A with G (c.286A>G).

Family history was positive for PED (for pedigree, see Figure 2). Her mother and grandmother also suffered from the disease with PED of the legs after prolonged walks or hiking. All affected family members started to suffer from PED with onset in childhood. The brother of the index patient has early-onset absence epilepsy (with onset in the third year of life) in addition to PED, and the grandmother suffered from Parkinson's disease and idiopathic/genetic generalized epilepsy with GTCS.

#### Genetic Testing and Variant Interpretation

In 2008, the first genetic test performing Sanger sequencing of *PNKD* and *SLC2A1* was negative. In December 2011, whole-exome sequencing revealed the missense variant c.287T>C in *SLC2A1* leading to an amino acid change from methionine to threonine (p.Met96Thr), which was reconfirmed by Sanger sequencing. The variant has been classified as pathogenic by MutationTaster and as probably damaging by PolyPhen-2. The amino acid position is located in the S3 helix and is conserved among several species (Figure 2). Evidence details have recently been uploaded to ClinVar (https://www.ncbi.nlm.nih.gov/clinvar/variation/847673/). The variant p.Met96Val (c.286A>G), which has been described as a *de novo* variant in a patient with GLUT1-DS (27), has been investigated recently showing a significant reduction of glucose uptake compared with average protein function (28). However, our variant p.Met96Thr has not been published or functionally analyzed before. According to ACMG criteria, the variant has been classified as a variant of uncertain significance until recently. We suggest that, with our additional evidence [cosegregation with disease in multiple affected family members in a gene definitely known to cause the disease (PP1 and PM5)], the variant can be reclassified as likely pathogenic (PM2, PM5, PP1, PP2, PP3).

### Individual #4: SLC2A1 p.Leu231Pro (c.692T>C)

#### Clinical Description

A 21-year-old man presented at our outpatient clinic for clarification of intermittent pain associated with cramps in the legs due to exertion, which he experienced the first time 1 year ago. In the beginning, symmetrical cramping pains in the legs had occurred in the afternoon, at the end of or after working as a warehouse keeper. When the symptoms were getting worse, the legs would turn inwards or forwards. Sometimes he noticed an indefinite feeling as a prodromal. When he sat down at the end of work, the symptoms eased after a certain time. Initially, L-dopa-sensitive dystonia was suspected by a practicing neurologist, but the symptoms did not improve by taking L-dopa. About 1 week after starting L-dopa treatment, he had his first and only GTCS. Immediately after the seizure, a cerebral CT scan and an EEG were performed, which were unremarkable. The individual had a history of early-onset absence epilepsy, which is why an antiepileptic treatment was started with valproic acid. There were no further seizures during the year until presentation, and also, the painful involuntary movements of the legs occurred less frequently.

Motor development was normal except for a circumscribed mild impairment of gross motor skills. He had mild psychomotor slowing and experienced difficulties in school with lack of concentration, but with support, he could complete secondary school. His maternal great-grandmother had a child with epilepsy (no further details available). The family history was otherwise negative for neurological diseases. On physical examination, mild gait insecurity was observed. The remaining neurological examination was normal.

At the age of 12 years, he underwent a 24-h EEG where generalized irregular 3/s spike-wave discharges with a maximal duration of 4 s had been recorded during sleep. There were no clinical absences during wakefulness. An MRI has never been performed.

#### Genetic Testing and Variant Interpretation

Whole-exome sequencing revealed the variant c.692T>C which results in a substitution of leucine with proline (p.Leu231Pro). There has been no contact with the father, which is why a segregation analysis was not possible. Amino acid alignment shows conservation of the amino acid among several species (human, mouse, rat, bovine, sheep, pig, rabbit, chicken). There is one publication of an individual with suspected GLUT1-DS which carried the same variant. Segregation analysis in this individual confirmed *de novo* status. She had predominant eyelid myoclonus with onset at the age of 3 months and mild intellectual disability, so that the phenotype of this individual differs significantly from ours, also indicating phenotypic heterogenicity (29). According to ACMG criteria, the variant is likely pathogenic (PM2, PM6, PP2, PP3, PP5), whereas criterion PM6 is based on the already published individual.

## Discussion

We here describe four individuals with PxD, three of whom bear newly identified likely pathogenic variants in known PxD genes (individual #1: *KCNA1*, p.Gly396Val; individual #2: *KCNA1*, p.Gly396Arg; and individual #3: *SLC2A1*, p.Met96Thr). The clinical presentation of the fourth individual expands the phenotypic spectrum of an *SLC2A1* variant previously associated only with GLUT1-DS (individual #4: *SLC2A1*, p.Leu231Pro).

The missense mutations of individual #1 and #2 (*KCNA1*; p.Gly396Val and p.Gly396Arg) further corroborate the recent findings of *KCNA1* as a causative gene for PxD since only two PKD families with an underlying *KCNA1* variant have been described so far (7, 8, 11). Moreover, these patients carry the first described *KCNA1* variants with PxD phenotype occurring *de novo*.

This evidence is urgently needed since only the index patient of the family carrying the variant p.Leu319Arg in *KCNA1* already published by Yin and colleagues (8) fulfilled the diagnostic criteria for PKD (3). Four other affected individuals with *KCNA1*-related PxD suffered from episodic dystonic attacks not fulfilling the criteria of PKD (3) because they were too long and not triggered by movement. The episodes were therefore termed “paroxysmal dyskinesia”—without further classification. Similar to our individual #2, the episodes were triggered by stress and anger (in our individual triggered by emotional stress). Given the long duration and/or the aforementioned triggering factors, one may suggest that there is a phenotypic overlap between PNKD in *KCNA1*-associated PxD. However, data on *KCNA1*-related PxD are only available from four different variants occurring in single families, so that closer assertions cannot be made yet.

*KCNA1* is commonly associated with episodic ataxia 1 (EA1) but shows a broad phenotypic spectrum including myokymia and hypomagnesemia without EA1, developmental and epileptic encephalopathy, and neuromyotonia (23). Up to 10% of patients with EA1 suffer from epilepsy (30), and myokymia and hyperthermia have been reported to co-occur in *KCNA1* mutation carriers as well. Therefore, it is not surprising that epilepsy has also been described to be a phenotypic feature of the known PxD-related *KCNA1* variants p.Leu319Arg and p.Asn255Lys (7, 8). Accordingly, individual #2 suffered also from epilepsy and myokymia, representing the diverse phenotypic spectrum of *KCNA1*-related disorders.

Currently, there is no targeted treatment available for *KCNA1*-associated disorders, which are mainly caused by a biophysical loss-of-function effect (23). The carbonic anhydrase inhibitor acetazolamide, which is often used in EA1 patients, seems to be effective in some patients, but there are still many patients suffering from uncontrolled disease course (31). Our individuals and the individuals of the affected family carrying the p.Leu319Arg variant responded well to the sodium channel blocker (SCB) oxcarbazepine, although seizure freedom was not reached. Unfortunately, treatment data of individuals with the p.Asn255Lys variant were not available, but individual #2 became free of attacks when another SCB—lacosamide—was added to oxcarbazepine. Consequently, we consider that the usage of SCBs in combination or as monotherapy (and in analogy with EA1) might be a good choice for acetazolamide-resistant patients or even as a first-line therapy in *KCNA1*-associated PxD. There have been first *in silico* approaches for a specific, causative treatment for *KCNA1*-related disorders with new substances (32) and different SCBs using a targeted “drug repurposing” approach (33). The biophysical ratio of a broadened shape of the action potential (AP) due to a reduced function of voltage-gated potassium tetramers, such as K_V_1.1 subunits, and a consequently affected repolarization phase (34) seems plausible, given the fact that SCBs could “correct” this broadened AP by very well-described effects, such as enhancing the inactivated state (35, 36), blocking the persistent sodium current [e.g., riluzole (37)], and reducing repetitive firing (38). Nevertheless, further studies in heterologous expression systems and neurons are necessary to enable individualized treatment.

The two families in our series with a variant in *SLC2A1* have PED and early-onset absence epilepsy or a positive family history of both. *SLC2A1* is known to be the major causative gene of the two diseases (39–41). We therefore think that the combination of the manifestations is highly suggestive for *SLC2A1* to be a candidate gene. The variant p.Met96Thr of individual #3 has not been described to date, but the evident cosegregation (Figure 2) increases the likelihood of pathogenicity of this variant. There have been functional measurements on a different variant at the same amino acid position (p.Met96Val), which has once been observed in a patient with GLUT1-DS (27, 28). A *Xenopus laevis* oocyte glucose uptake assay showed a significantly marked reduction of glucose uptake compared with the average protein function indicating pathogenicity. The variant with a valine substitution though has been found among 3 of 12,332 individuals of Finnish ancestry so that the likelihood of pathogenicity of this variant (p.Met96Val) becomes more disputable (21, 28).

Our case series has major limitations as we do only have single individuals or families carrying the presented variants and we do not have functional data to support our hypotheses. We know that the variants can only be classified as likely pathogenic and therefore leave room for some uncertainty. Also, we could not reliably retrace why the *SLC2A1* variant of individual #3 was missed in the first sequencing approach *via* single-gene sequencing. However, we believe that in conjunction with the literature on related variants and their phenotypes, we increase the evidence of pathogenicity of the detected variants and give an overview of the current knowledge about them. Furthermore, functional characterizations of three other variants in homologous channels at the same position but different amino acids revealed a clear loss of function (see results). We want to highlight that two of our four described variants (*KCNA1* p.Gly396Val; *SLC2A1* p.Met96Thr) have been identified by reanalysis of existing genetic information. Therefore, reanalysis of existing genomic data may be considered, especially if the phenotype is highly suggestive for a specific gene or genetic phenotype. We encourage clinicians and researchers to share clinical information of individuals carrying rare or novel likely pathogenic or pathogenic variants, which might lead to a better understanding of the disease mechanism.

## Data Availability Statement

The original contributions presented in the study are publicly available. This data can be found at ClinVar with the following accession numbers: NM_000217.3(KCNA1):c.1187G>T SCV001623013; NM_000217.3(KCNA1):c.1186G>C SCV001623014; NM_006516.4(SLC2A1):c.287T>C SCV001623015 and NM_006516.4(SLC2A1):c.692T>C SCV001623016.

## Ethics Statement

The studies involving human participants were reviewed and approved by Ethics Committee at the Medical Faculty of the University of Tuebingen and the Comite de Etica de Investicacion con medicamentos La Fe. Written informed consent to participate in this study was provided by the participants' legal guardian/next of kin. Written informed consent was obtained from the individual(s), and minor(s)' legal guardian/next of kin, for the publication of any potentially identifiable images or data included in this article.

## Author Contributions

JKe, LL, AM, AVM, and FM recruited and phenotyped the patients. JKr, MK, JKe, AVM, FM, and SL analyzed and interpreted the genetic data. JKe, JKr, and SL wrote the manuscript. All authors read, revised, and approved the manuscript.

## Conflict of Interest

The authors declare that the research was conducted in the absence of any commercial or financial relationships that could be construed as a potential conflict of interest.
